# Bisphenol A decreases peripheral insulin sensitivity in normal weight adults: a double-blind randomized controlled trial

**DOI:** 10.1210/clinem/dgag124

**Published:** 2026-03-20

**Authors:** Adam D Seal, Suzanne Phelan, Steven K Malin, Andrew Schaffner, Hannah B Gaydos, Ryan Hubbard, Kelly A Bennion, Sarah Keadle, Jane Nakamura, Alia Ortiz, Clara McMahon, Todd A Hagobian

**Affiliations:** Center for Health Research, California Polytechnic State University, San Luis Obispo, CA 93407, USA; Center for Health Research, California Polytechnic State University, San Luis Obispo, CA 93407, USA; Department of Kinesiology and Health, Rutgers University, New Brunswick, NJ 08901, USA; Department of Statistics, California Polytechnic State University, San Luis Obispo, CA 93407, USA; Center for Health Research, California Polytechnic State University, San Luis Obispo, CA 93407, USA; Center for Health Research, California Polytechnic State University, San Luis Obispo, CA 93407, USA; Department of Psychology and Child Development, California Polytechnic State University, San Luis Obispo, CA 93407, USA; Center for Health Research, California Polytechnic State University, San Luis Obispo, CA 93407, USA; School of Kinesiology, University of Michigan, Ann Arbor, MI 48109, USA; Center for Health Research, California Polytechnic State University, San Luis Obispo, CA 93407, USA; Center for Health Research, California Polytechnic State University, San Luis Obispo, CA 93407, USA; Center for Health Research, California Polytechnic State University, San Luis Obispo, CA 93407, USA

**Keywords:** bisphenol A, insulin sensitivity, type 2 diabetes

## Abstract

**Context:**

Bisphenol A (BPA) is a synthetic chemical used in consumer goods that has been linked to type 2 diabetes in observational studies. No human experimental studies have examined whether BPA reduces peripheral insulin sensitivity.

**Purpose:**

To determine the effects of oral BPA administration on peripheral insulin sensitivity over 5 days.

**Methods:**

Forty sedentary, but otherwise healthy adults (22 F, 18 M; 21.3 ± 2.1 year; 22.1 ± 2.3 kg/m^2^; 85% non-Hispanic White) completed a 2-day baseline energy-balanced diet low in BPA during which urine, blood, and peripheral insulin sensitivity via 120 minutes euglycemic hyperinsulinemic clamp technique (40 mU/m^2^/min; 90 mg/dL) were assessed. In a double-blind fashion, participants were randomly assigned to receive 5 days of oral BPA administration at 50 µg/kg body weight (BPA-50) or placebo (PL). All participants were fed the same energy-balanced diet. Outcomes were assessed using a two-way repeated-measures ANOVA adjusted for baseline sex, BMI, physical activity, and ethnicity.

**Results:**

After treatment, urine BPA levels were significantly higher in BPA-50 compared to PL (+ 268 700 ± 63 983 vs PL: + 8893 ± 6786 pg/mL, *P* = .009). After treatment, body weight and fasting glucose were not statistically different between PL and BPA-50 (*P* > .05). However, BPA significantly decreased peripheral insulin sensitivity by 0.02 ± 0.01 mg/kg/min/uU/mL compared with an increase observed following PL by 0.02 ± 0.01 mg/kg/min/uU/mL (*P* = .01).

**Conclusion:**

BPA administration decreased peripheral insulin sensitivity after 5 days. These data provide the first experimental evidence in humans that BPA administration may reduce insulin sensitivity.

Bisphenol A (BPA) is a widely produced synthetic chemical used in various consumer and industrial products (1). Human exposure to BPA is widespread, as NHANES data suggest that 93% of the population has detectable BPA in their urine (2). Observational studies have shown positive associations between urinary BPA concentrations and incidence of type 2 diabetes (3), prediabetes (4), insulin resistance (5), and hemoglobin A1c (6), suggesting that BPA may have some role in type 2 diabetes risk. The mechanisms linking BPA exposure to type 2 diabetes risk remain unclear; however, animal and *in vitro* data suggest that BPA has estrogenic activity and disrupts several pathways in the pathogenesis of type 2 diabetes, including dysregulation of glucose metabolism (7), decreased insulin sensitivity (8, 9), and altered pancreatic beta*-*cell and hepatic cell function (10).

Surprisingly, few published studies have examined the direct effects of BPA administration on human diabetes risk. Our previous pilot study (11) and Stahlhut et al (12). showed that a single BPA administration altered glucose and insulin responses during an oral glucose tolerance test in healthy adults. Whether the dysregulation in glucose metabolism was due to insulin sensitivity could not be directly determined. Thus, to accurately determine the direct effects of BPA on insulin sensitivity, we sought to conduct a controlled experimental design over several days using gold-standard measures. The primary purpose of this study was to determine whether oral administration of BPA over 5 days, consistent with the US EPA reference dose (13) of 50 μg/kg body weight (BPA-50), had an independent effect on peripheral insulin sensitivity in adults with normal body weight. We hypothesized that the BPA-50, compared to placebo (PL), would decrease peripheral insulin sensitivity. Secondarily, we examined other endocrine factors and indices of insulin resistance (C-peptide, free fatty acids, 17β-estradiol, HOMA-IR, Adipose-IR), as well as psychosocial factors (mood) and behavioral factors (sleep, physical activity), to gain insight into known contributors to changes in peripheral insulin sensitivity and factors known to be affected by BPA exposure.

## Materials and methods

### Participants

Forty adults (22 F, 18 M; 85% non-Hispanic White) with a mean age of 21.3 (0.3) year and a mean BMI of 22.1 (0.4) kg/m^2^ (Table 1) were recruited from the San Luis Obispo, CA area through flyers and university advertisements from January 2020 to May 2023. Eligibility included BMI 18.5 to 25 kg/m^2^, 18 to 40 years old, and hemoglobin A1c < 5.7% (assessed by finger stick; PTS Diagnostics, Whitestown, IN, USA). Participants were required to be non-smoking, engage in <150 minutes/week of aerobic exercise, not on any prescribed or over-the-counter medications, be nonpregnant or not trying to become pregnant, free from neurological disorders, and be free of type 1 or type 2 diabetes and other chronic diseases as assessed by a health history questionnaire. This study was approved by the Institutional Review Board at California Polytechnic State University (2018-149-CP) before data collection and conducted in accordance with the Declaration of Helsinki. Participants gave written and verbal consent and were compensated $100 for completing preliminary measures and an additional $400 for completing all measures. The study was registered on ClinicalTrials.gov (NCT03771066).

**Table 1:** Participant characteristics (*n* = 40)

	Placebo	BPA-50	All	*P*-value
Female sex, *n* (%)	12 (55)	10 (45)	22 (55)	.77
Hispanic Ethnicity, *n* (%)	5 (25)	3 (15)	8 (20)	.53
Age (years)	21.2 (0.3)	21.3 (0.6)	21.3 (0.3)	.53
Body weight (kg)	66.7 (2.2)	66.7 (2.9)	66.7 (1.8)	.61
PA (hours/week)	5.7 (0.2)	5.5 (0.2)	5.6 (0.1)	.91
BMI (kg/m^2^)	22.4 (0.5)	22.1 (0.5)	22.2 (0.4)	.98
REE (kcal/day)	1626 (49.1)	1638.3 (97.7)	1632 (52.5)	.70
HbA1c (%)	4.7 (0.1)	4.8 (0.1)	4.8 (0.1)	.96
Fasting glucose (mg/dL)	95.6 (2.0)	92.3 (2.4)	93.9 (1.6)	.20

Values represent mean (SE) unless otherwise noted.

Abbreviations: BMI, body mass index; PA, self-reported physical activity; REE, resting energy expenditure.

### Experimental protocol

**Figure 1:**
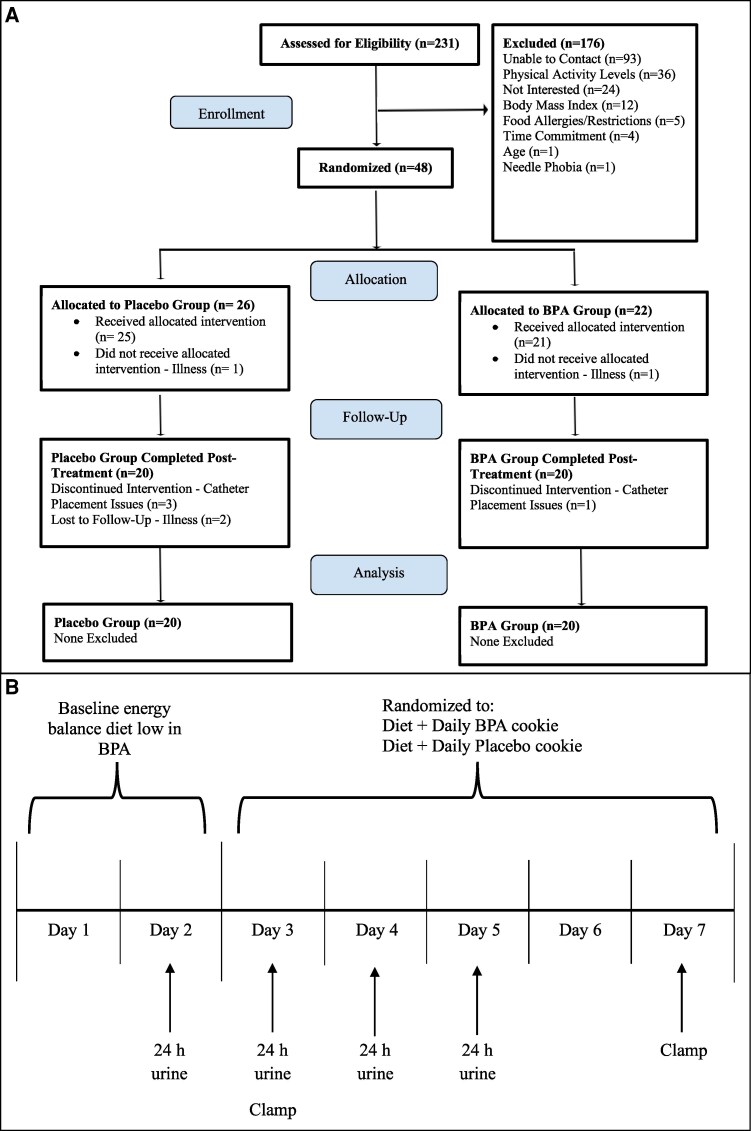
(A) CONSORT diagram of progress through the phases of the parallel randomized trial (B) overview of experimental protocol.

This study adhered to the Consolidated Standards of Reporting Trials (CONSORT) guidelines for parallel-group randomized trials (14). Figure 1A shows the CONSORT diagram of the progress through the phases of the parallel randomized trial. After eligibility was determined and consent was obtained, participants completed a demographic questionnaire assessing age, sex, ethnicity, and physical activity (International Physical Activity Questionnaire) (15). Participants were given a GeneActiv physical activity monitor (Activinsights, Kimbolton, England) to wear throughout the study to assess activity levels. Weight was measured using a digital scale (Continental Scale Corporation, Bridgeview, IL, USA), and height was recorded using a stadiometer (Ellard Instrumentation LTD., Monroe, WA).

After preliminary measures, resting metabolic rate was calculated using the Harris–Benedict equation (16), and an appropriate activity factor was applied to determine an energy-balanced diet. The macronutrient composition was consistent across all participant diets, with approximately 55% carbohydrate, 30% fat, and 15% protein. All food was sourced from local grocery stores in San Luis Obispo, CA, and our laboratory had previously tested the food and water to ensure low levels of BPA and bisphenol S (BPS) (<0.20 ng/g fresh weight) (17). Participants were provided with BPA-free commercial water (Boxed Water Inc.) and were asked to track their total water intake on a provided form. All BPA and BPS measurements were below 0.02 ng/g fresh weight. No other chemical agents were tested. All foods and beverages were stored in BPA-free glass containers and handled with BPA-free stainless steel utensils, as indicated by the manufacturing labels.

Participants then completed a 2-day baseline period before the first insulin sensitivity test. During this 2-day period, participants were fed an energy balance diet. 24-hour urine, sleep quality using a visual analog scale (Leeds Sleep Questionnaire) (18), mood (Profile of Mood States) (19), and gastrointestinal distress (20) information were collected. Physical activity was monitored using a wrist-worn accelerometer device (GENEActiv, ActivInsights Ltd., Kimbolton, England). The GGIR package version 3.3, with the Hildebrand cut-point (93.2 mg), was used to calculate minutes of moderate-to-vigorous physical activity from the GENEactiv device (21, 22). Participants were then randomized in a double-blind fashion (blocked by sex and ethnicity) to either administration of placebo (PL) or BPA (described below for dose details) for 5 days. The study statistician used a computer-generated randomization scheme without contact with study participants. Participants resumed the energy balance diet low in bisphenols (23) throughout the treatment period, during which 24-hour urine samples, fasting blood samples, physical activity, and peripheral insulin sensitivity were repeatedly assessed (Fig. 1B).

### Urine collection and analysis

At baseline and during the 5-day treatment period, urine was collected every 6 hours for 24 hours. Participants voided into a container kept in a cooler. After measuring urine volume with a graduated cylinder, 15 mL was aliquoted into 3 separate BPA-free polypropylene tubes at each 6-hour interval and stored at −80 °C. All urine samples were analyzed for free BPA concentrations unadjusted for creatinine using a commercially available enzyme-linked immunosorbent assay (ELISA) (Detroit R&D Inc., Detroit, MI, Catalog # BPA1, RRID: AB 2754549).

### Fasting blood and peripheral insulin sensitivity

At baseline and day 5 of the treatment period, participants arrived at the laboratory at 0700 after a 10-hour overnight fast. Fasting hormones and endocrine factors linked to the pathogenesis of type 2 diabetes, including glucose, insulin, C-peptide, free fatty acids (FFA), and 17-beta-estradiol, were collected in the appropriate vacutainer. Peripheral insulin sensitivity was then assessed using the euglycemic hyperinsulinemic clamp technique described in previous studies (24, 25). Two intravenous infusions were started with a peristaltic pump (Harvard Apparatus, Holliston, MA, USA): (1) primed 250 mU/m^−2^ and then constant 40 mU/m^−2^/min^−1^ of insulin diluted in saline containing 4% of the participant's blood (26) and (2) a variable infusion of a 20% glucose saline solution adjusted to maintain plasma glucose at 90 mg/dL and continued for 120 minutes. Plasma glucose analysis was performed every 5 minutes using the glucose oxidation method (YSI Inc., Yellow Springs, OH, USA). Circulating insulin, C-peptide, and FFA were also assessed at 90, 105, and 120 minutes of the clamp and averaged to approximate a steady state. Glucose infusion rates were averaged during the last 30 minutes of the clamp, as this was considered the time period to achieve steady-state. Peripheral insulin sensitivity was defined as the glucose infusion rate divided by the steady-state plasma insulin concentration. Homeostatic model assessment of insulin resistance (HOMA-IR), adipose tissue insulin resistance (Adipose-IR), and insulin-stimulated FFA (lipolysis) suppression (FFA_suppression_) were also calculated as previously described (27, 28). Blood samples were centrifuged at 4 °C for 15 minutes at 3000 RPM and stored at −80 °C until subsequent analysis. Plasma insulin (Merck Inc., Darmstadt, Germany, Catalog # EZHI-14K, RRID: AB_2800327), C-peptide (Merck Inc., Catalog # EZHCP-20K, RRID: AB_2800332), FFAs (Antibodies-online.com Inc., Pottstown, PA, USA, Catalog # ABIN5708933, RRID: AB_10769133), and 17-beta estradiol (Antibodies-online.com Inc., Catalog # ABIN284395, RRID: AB_10769133) were analyzed in duplicate using ELISAs. If the CV between duplicate measurements exceeded 10%, a third measurement was analyzed.

### BPA administration

Oral BPA at 50 µg/kg of body weight or PL was administered on a vanilla wafer cookie for the 5 days of treatment, similar to our preliminary and previous pharmacokinetic studies (11, 29, 30). A single dosing solution, relative to participant body weight, was created by dissolving d6-BPA (C/D/*N* Isotopes, Pointe-Claire, Quebec) in absolute 95% ethanol (Acros Organics, Janssen Pharmaceutical, Belgium). For the placebo, d6-BPA was not included in the ethanol solution. A research assistant not involved in any other aspect of this study made the dosing solutions. Approximately 1 mL aliquots were passed twice through a sterile microfilter to remove bacteria and then placed onto a vanilla wafer cookie (20 grams). The ethanol was allowed to dry for 8 hours before daily consumption. The vanilla wafer cookies with BPA-50 and PL looked, weighed, and tasted identical. All study personnel and participants were blinded from the random assignment to either PL or BPA-50, except the principal investigator, who had no contact with any participants. Each morning during the 5-day treatment period, a research assistant observed participants consuming the vanilla wafer cookie with BPA-50 or PL. The final dose was administered one hour before the start of the final euglycemic hyperinsulinemic clamp.

### Statistical analysis

JMP Pro 17.0.0 (SAS Institute, Cary, NC) was used for all statistical analyses. Summary statistics and figures report the mean with SEM unless otherwise noted. To ensure data quality, analyses were conducted for each study variable to identify outliers and verify that reported values fell within logical ranges. All nondetectable concentrations of BPA, plasma insulin, C-peptide, and FFA were assigned a single imputation at the lower limit of detection described in kit protocols. Utilizing a single imputation at the lower limit of detection when fewer than 30% of samples yield nondetectable concentrations, as in the present study, has been shown to prevent bias in analyzing biomarker data (31). A two-way repeated-measures ANOVA was used to examine the effects of group, time, and the interaction between group and time on peripheral insulin sensitivity, while adjusting for covariates including baseline insulin sensitivity, BMI, weekly minutes of moderate-to-vigorous physical activity, sex, and ethnicity (Hispanic or non-Hispanic). Another two-way repeated-measures ANOVA with the same covariates was used to examine the effects of group, time, and group × time interaction on steady-state glucose infusion rate, without adjustment for insulin. A repeated-measures ANOVA was also used to assess differences in HOMA-IR, C-peptide-to-glucose ratio, adipose-IR, FFA _suppression_, plasma insulin, C-peptide, and FFA levels, as well as mood, gastrointestinal distress, and sleep. A paired two-tailed *t*-test was used to evaluate urinary BPA differences between the BPA-50 and PL groups, as well as to examine demographic differences and caloric and macronutrient intake between the baseline and 5-day treatment periods. A significance level of *α* ≤ .05 was used.

Because no previous experimental studies had evaluated the direct effects of BPA consumption on peripheral insulin sensitivity over several days in humans, the power calculation and sample size for this study were based on the repeated-measures analysis of metabolized glucose from the original Defronzo and Matsuda euglycemic hyperinsulinemic clamp paper, which accounted for the observed individual variation of 20% to 25% in insulin sensitivity (32). The SD of the differences in the repeated-measures design was 0.448 mg/kg⋅min. This is equivalent to a medium effect size. With 40 total participants at baseline, assuming 10% lost to follow-up at the final assessment (*N* = 36), we had 80% power to detect a 0.52 difference in glucose infusion rate (mg/kg·min) between BPA-50 and PL using an *α* = .05 and a two-sided test of significance.

## Results

**Figure 2:**
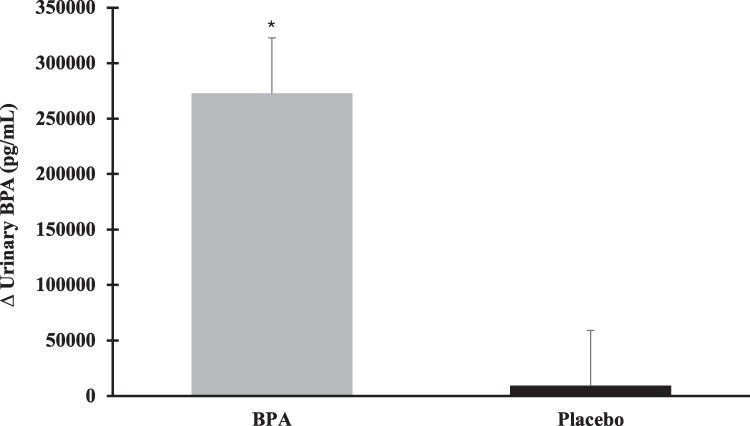
Change in urinary BPA concentrations in BPA-50 and PL.

There were no baseline statistical differences in urinary BPA between BPA-50 and PL (BPA-50: 2402 (554) vs PL: 1433 (485) pg/mL, *P* = .198). As expected, urinary BPA increased in the BPA-50 group from the baseline to the end of the 5-day treatment period compared with PL (BPA-50: + 268 700 ± 63 983 vs PL: + 8893 ± 6786 pg/mL, *P* = .009, Fig. 2). This suggests that urinary BPA concentration was successfully increased by oral administration of BPA at 50 g/kg body weight.

**Figure 3:**
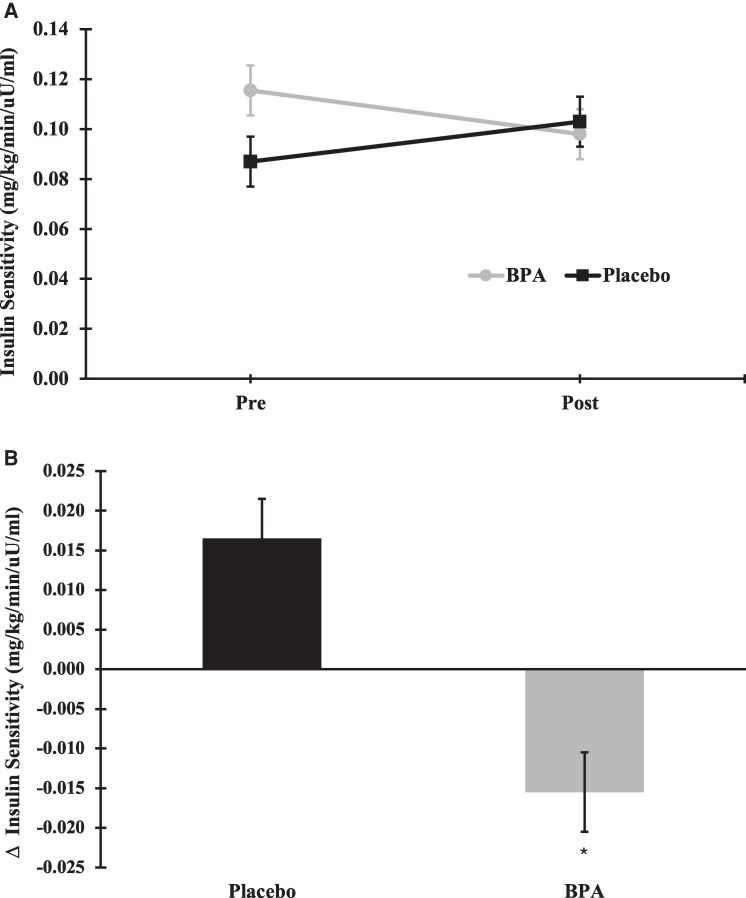
(A) Insulin sensitivity in BPA-50 and PL between baseline and post-treatment clamps (B) change in insulin sensitivity in PL and BPA-50.

After 5 days of treatment, peripheral insulin sensitivity was significantly decreased in BPA-50 by 0.02 mg/kg/min/uU/mL compared with a slight rise after PL (group × time interaction, F (1, 37.9) = 6.98, *P* = .012; Fig. 3A). There was a significant main effect of group (F = 6.12, *P* = .02, Fig. 3B) and sex (males: 0.11 vs females: 0.09 mg/kg/min/uU/mL, F = 4.72, *P* = .04) on the difference in insulin sensitivity comparing baseline and 5-day treatment clamps. After 5 days of treatment, there were no between-group differences in steady-state glucose infusion rate (*P* = .21). In PL, 14 participants' insulin sensitivity improved or remained unchanged, while 6 participants' insulin sensitivity worsened. In BPA-50, 6 participants' insulin sensitivity improved, while 14 participants' insulin sensitivity worsened or remained unchanged.

**Figure 4:**
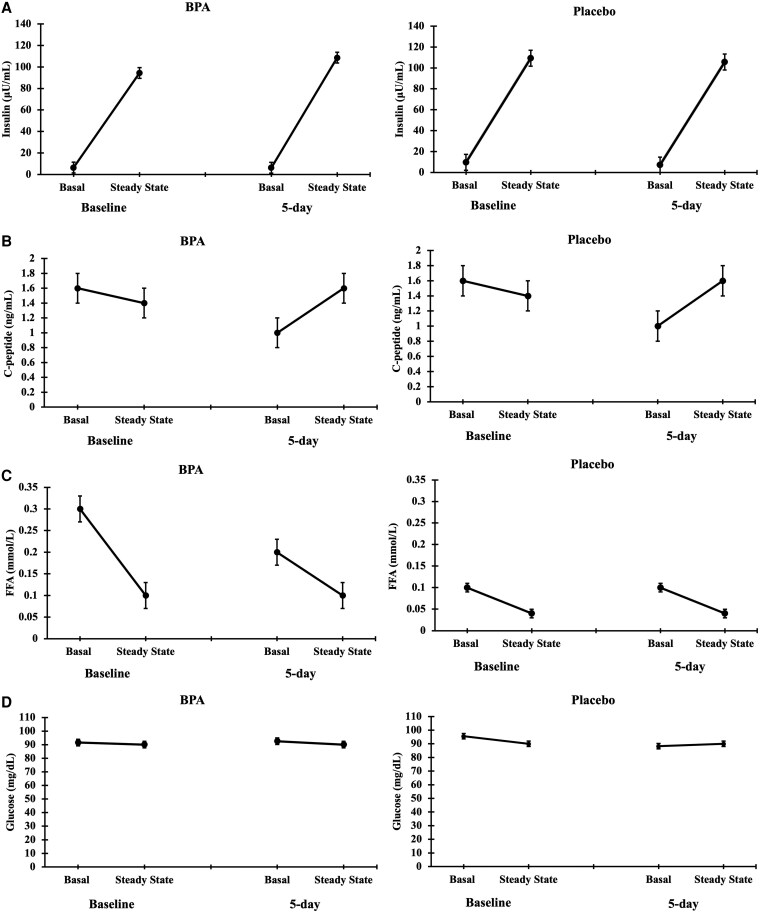
(A) Insulin, (B) C-peptide, (C) FFA, and (D) glucose during clamps for BPA-50 and PL.

There were no statistical differences observed between BPA-50 and PL for HOMA-IR (*P* = .14), adipose-IR (*P* = .86), C-peptide to glucose ratio (*P* = .40), or FFA_suppression_ (*P* = .53) after treatment (Table 2). There were no statistical differences between BPA-50 and PL from basal to steady-state during either the baseline or 5-day euglycemic hyperinsulinemic clamp technique in insulin (*P* = .26), C-peptide (*P* = .38), FFA (*P* = .53), glucose (0.09) (Fig. 4), or estradiol (*P* = .46).

**Table 2:** Insulin metabolism indices

	Placebo		BPA-50		*P*-value
	Baseline	5-day	Baseline	5-day	
HOMA-IR	2.28 (0.4)	1.54 (0.2)	1.45 (0.2)	1.49 (0.4)	.14
CP/G	1.02 (0.1)	1.12 (0.1)	1.10 (0.1)	1.00 (0.1)	.40
Adipose-IR	1.23 (0.6)	0.83 (0.4)	1.45 (0.3)	1.23 (0.6)	.86
FFA_suppression_ (%)	47.5 (9.2)	51.2 (8.7)	61.1 (5.8)	57.6 (7.7)	.53
Glucose (mg/dL)	95.6 (2.0)	88.3 (1.9)	91.5 (2.4)	92.5 (1.8)	.09

Values represent mean (SE).

Abbreviations: CP/G, C-peptide to glucose ratio × 100; FFA suppression, insulin-stimulated free fatty acid suppression.

Similarly, from baseline to 5-day treatment, there was no statistical evidence of change in body weight (*P* = .61) or fasting blood glucose (*P* = .09) in either PL or BPA-50. There were also no statistically significant differences in caloric intake (*P* = .57), water intake (*P* = .33), or macronutrient intake (*P* > .05 for fat, carbohydrate, and protein), minutes of moderate to vigorous physical activity (*P* = .22), sleep (*P* = .97), mood (*P* = .19), or GI distress (*P* = .61) between baseline and the 5-day treatment between PL or BPA-50 (Table 3).

**Table 3:** Physical activity, mood, GI distress, sleep, energy intake

	Placebo		BPA-50		*P*-value
	Baseline	5 day	Baseline	5 day	Group × time
MVPA (min/day)	24.2 (7.1)	23.6 (4.6)	34.1 (8.1)	18.1 (4.8)	.22
Mood (a.u.)	7.2 (2.0)	8.2 (1.7)	10.1 (1.7)	6.7 (1.0)	.19
GI distress (a.u.)	2.7 (0.9)	1.3 (0.5)	4.7 (1.5)	4.6 (1.3)	.61
Sleep (mm)	52.3 (2.1)	51.1 (2.1)	51.9 (2.4)	50.6 (2.4)	.97
Energy intake (kcals)	2156 (62)	2301 (61)	2273 (63)	2331 (105)	.57
CHO, g (%)	314 (58)	323 (56)	329 (58)	338 (58)	.99
Fat, g (%)	81 (34)	83 (32)	81 (32)	85 (33)	.70
PRO, g (%)	93 (16)	94 (16)	93 (16)	94 (16)	.60

Values represent mean (SE).

Abbreviations: CHO, carbohydrate; MVPA, moderate to vigorous physical activity as measured by GENEActiv; PRO, protein.

## Discussion

The primary finding was that BPA administration at the US EPA-approved reference dose significantly decreased peripheral insulin sensitivity in normal-weight adults. In contrast, no significant changes were observed in fasting glucose or hormones, behavioral measures, or psychological parameters. Regardless, findings align with prior animal studies (9, 33) and provide the first experimental evidence in humans that short-term oral BPA administration may contribute to the development of insulin resistance. Thus, BPA at the US EPA-approved safe dose may be an environmental factor in consumer and manufacturing goods that may affect insulin sensitivity.

Previous evidence linking BPA exposure to insulin resistance has been mainly associative. Epidemiological studies have reported associations between urinary BPA and markers of insulin resistance and diabetes across the human lifespan (34, 35). Surprisingly, few experimental studies have examined the direct effects of oral BPA administration on indices of insulin resistance and glucose metabolism. Both our previous pilot study (11) and Stahlhut et al (12). showed that a single oral dose of BPA at 50 µg/kg acutely lowered insulin and glucose responses. However, this prior work was unable to discern the direct effects of insulin sensitivity from those of insulin secretion, given that an oral glucose tolerance test was used. The present study extends these findings by showing that BPA administration over 5 days reduced peripheral insulin sensitivity by approximately 9% using the euglycemic-hyperinsulinemic technique. While we did not use stable isotopes to disentangle skeletal muscle from hepatic glucose production, we observed no difference in HOMA-IR, a commonly used index of hepatic insulin resistance. This suggests that BPA may have interfered with insulin-stimulated glucose metabolism in skeletal muscle. Interestingly, we observed a trend for fasting glucose to rise by approximately 1 mg/dL following BPA, compared with a nearly 7 mg/dL drop after PL. A possible reason is that fasting insulin levels were slightly lower after BPA, consistent with the literature suggesting that BPA may impair beta-cell function (36). In the present study, we also observed no change in fasting C-peptide levels. Work using hyperglycemic clamps and/or mixed-meal tolerance tests would be necessary to determine whether BPA alters pancreatic insulin secretion. Regardless, we did not observe changes in adipose-IR or FFA suppression between BPA and PL treatments, suggesting adipose insulin sensitivity was unaffected by BPA in this study. Lastly, we did not observe changes in 17β-estradiol across treatments, indicating that estrogen is unlikely to explain the reduction in peripheral insulin sensitivity after BPA in the present short-term study. Future studies incorporating complementary gold-standard methodologies, such as stable isotope infusion to assess hepatic glucose production and lipid metabolism, are needed to confirm and explain these findings and elucidate the pathways through which BPA may impair glucose regulation.

To the best of our knowledge, these results provide the first published experimental evidence in humans that short-term oral BPA administration may impair insulin sensitivity. In the present study, a reduction in peripheral insulin sensitivity was observed at the US EPA reference dose for BPA, a level designated as the “safe” lifetime exposure threshold (13). Previous human studies have evaluated glucose metabolism and pharmacokinetics of a single oral BPA administration, reporting a maximum blood concentration of 15.9 µg/L. In contrast, the present study observed substantially higher urinary BPA concentrations (269 µg/L) following 5 days of repeated oral exposure. Importantly, no gastrointestinal distress (as assessed by questionnaire) or unintended adverse events (per CONSORT guidelines) were reported. These findings suggest that short-term oral BPA administration at the applied dose is feasible and well-tolerated in healthy young adults.

Previous studies have reported that BPA exposure is associated with adverse effects on mood (37) and sleep quality (38). However, experimental evidence directly testing these relationships in humans remains limited. In the present study, no significant differences in mood or sleep were observed between the placebo and BPA-50 groups when assessed by validated questionnaires. These findings suggest that short-term BPA administration at the EPA reference dose may not have an acute influence on self-reported psychological or sleep-related outcomes. Nevertheless, future studies employing objective measures such as polysomnography, along with more prolonged exposure durations, are warranted to more comprehensively evaluate the potential impact of BPA on sleep and mood regulation.

This study has several notable strengths. Strengths include the rigorous design, which is a double-blind, placebo-controlled, randomized clinical trial conducted in accordance with CONSORT guidelines, and the use of the hyperinsulinemic–euglycemic clamp, the gold-standard method for assessing peripheral insulin sensitivity. Participants consumed only foods prescreened by our laboratory, which were low in BPA and BPS (23), and strict protocols were implemented to minimize background BPA exposure. Potential confounding factors—including physical activity, caloric and macronutrient intake, sleep, and mood—were also closely monitored, thereby isolating the effect of BPA on insulin sensitivity. However, this study has limitations that should be acknowledged. Although randomization was stratified by sex and ethnicity, the study population consisted mainly of young, college-aged adults recruited through convenience sampling. This limits the generalizability of the findings to broader populations with greater demographic diversity. Moreover, it is not clear how BPA interacts with people who have obesity or prediabetes/type 2 diabetes, and this warrants attention. It was somewhat surprising that insulin sensitivity improved in the PL group. We suspect that the consumption of the fresh-food diet designed for weight maintenance in our participants randomized to PL may have provided some benefit compared with their habitual eating habits, as suggested in previous studies, given that these participants were primarily young college adults (39). Regardless, both groups received isocaloric diets with comparable macronutrients, implying that any diet-related improvements would have occurred equally across conditions. Finally, future studies should investigate whether reduced peripheral insulin sensitivity would improve after cessation of BPA consumption.

In conclusion, 5 days of oral BPA administration at the US EPA reference dose reduced peripheral insulin sensitivity in adults with normal weight. These results carry important clinical and public health implications, suggesting that BPA exposure, even at doses currently considered “safe,” may contribute to the development of insulin resistance. When considered alongside prior epidemiological (40), animal (36), and human studies (11, 12), the present findings add to the growing body of evidence that regulatory agencies should critically reevaluate the established reference dose for BPA.

## Data Availability

Some or all datasets generated during and/or analyzed during the current study are not publicly available but are available from the corresponding author on reasonable request.

## Clinical Trial Information

NCT03771066.
